# Can Anxiety Affect Dermographism and Atopy Test Outcomes?

**DOI:** 10.7759/cureus.21994

**Published:** 2022-02-07

**Authors:** Nurullah Yekta Akcam, Güzin Özden

**Affiliations:** 1 Allergy and Immunology, Mersin City Training and Research Hospital, Mersin, TUR; 2 Allergy and Immunology, Adana City Training and Research Hospital, Adana, TUR

**Keywords:** prick test, phadiatop, dermographism, anxiety, atopy

## Abstract

Aim: The effect of anxiety on dermographism and atopy test results had never been elaborated. Factors that may affect cutaneous reactivity in skin tests should be determined to improve the accuracy and reliability of results. Age, sex, dermographism, race, seasons of the year, circadian rhythm, and some drugs have been shown to affect skin test results. The aim of this study was to investigate the effect of anxiety on dermographism and atopy tests.

Method: According to Beck Anxiety Scale, 101 individuals with high anxiety levels were compared with the other 101 individuals with an anxiety level of 7 and below. Skin prick test response, presence of phadiatope, and dermographism were evaluated in all participants.

Results: There were 41 (40.6%) and 34 patients (33.7%) with a Beck anxiety score >7 who tested positive in the skin test and Phadiatop, respectively. Moreover, 47 (46.5%) and 42 patients (41.6%) in the control group had positive results in the skin test and Phadiatop, respectively, and there was no statistical difference between the variables (p > 0.05). Dermographism was present in 37 (36.6%) and 30 patients (29.7%) in the study and control groups, respectively. According to Beck anxiety scores, there was no statistically significant difference between the groups in terms of the presence of dermographism, skin prick test positivity, and Phadiatop positivity (p = 0.078, p = 0.395, p = 0.245, respectively). Symptomatic dermographism was found more frequently in the anxiety group (p < 0.05).

Conclusion: In our study, anxiety does not affect atopy test results. Although anxiety levels did not affect the atopy test results, there was a relationship between anxiety levels and dermographism.

## Introduction

Atopy is a state in which there is increased production of immunoglobulin E (IgE) antibodies to allergens [1]. Atopy is used to describe IgE-mediated allergic diseases that have a genetic predisposition such as asthma, allergic rhinoconjunctivitis, and atopic dermatitis [2].

Atopic diseases are early-type IgE-mediated immune reactions, and diagnosing allergies requires a thorough clinical history, skin tests, specific IgE measurements, and in some cases, provocation tests [3]. Skin tests are the recommended initial diagnostic method due to their high sensitivity and reliability, cost-effectiveness, and ease of application [4-5]. In predisposed individuals, dermally administered allergens (skin prick test) cross-link with IgE on the surfaces of mast cells and stimulate them, which releases mediators that cause edema and erythema of the surrounding skin [6]. The primary mediators of erythema and edema are histamine and cellular and neurogenic structures (substance P, calcitonin gene-dependent peptide), and neuropeptides, such as neurokinin A/B, can also contribute to this early reaction [7]. Histamine triggers the release of substance P via the axonal reflex, which increases the early reaction (Lewis’s triple response) by causing mast cells in the skin to release histamine [8]. Mast cells can be stimulated by both immunological factors as well as non-immunological factors such as physical trauma. The response to physical trauma from the skin is known as the triple response of Lewis [9-10]. An exaggerated triple response of Lewis, called dermographism, is characterized by a magnified wheal-and-flare reaction [11]. In skin tests, physical trauma during epicutaneous application causes the skin to swell, resulting in redness of the skin even without sensitization in individuals with dermographism. This reaction could limit the diagnostic value of skin testing [12]. “Dermatographism,” which literally means “writing on the skin,” is the most common type of inducible physical urticaria, occurring in 1.5%-5% of the population [13].

Factors that could affect cutaneous reactivity in skin tests are evaluated using negative and positive controls. The quality of the diluents, areas where the allergen is applied, age, race, seasonal factors, and some drugs have been shown to affect test results [14-15].

 Measuring the specific IgE in the blood (Phadiatop) is a time-consuming and expensive method, although it is advantageous in some patients, such as in those who use anti-histaminic drugs, those with severe dermographism, and generalized skin disease, or those with severe reactions to skin tests. A well-established correlation exists between Phadiatop and skin prick test results [15]; however, skin tests are more sensitive and specific [14-15].

 Anxiety disorders are the most common type of mental health disorder in the general population [16-17]. Anxiety is a specific condition wherein feelings of anxiety and fear accompany somatic symptoms due to overstimulation of the autonomic nervous system [18]. Some studies have reported that anxiety and psychological stress can influence allergy-related immune functions [19-20].

 The immune and nervous systems have a bilateral relationship, and this interaction occurs through nerves as well as cytokines and neuropeptides secreted locally from the skin. The nervous system modulates the immune system via neurotransmitters (acetylcholine noradrenaline, serotonin, histamine), neuropeptides [adrenocorticotropic hormone (ACTH), prolactin, vasopressin, bradykinin, somatostatin, vasoactive intestinal peptide (VIP), neuropeptide Y], neurological growth factors (neuron growth factor), and hormones (adrenaline and corticoids), and the immune system influences nervous system functions through a variety of molecules, including cytokines [tumor necrosis factor alpha (TNFα) and transforming growth factor beta (TGFb)], chemokines (interferon), and nitric oxide [19, 21-22]. Proinflammatory cytokines in individuals experiencing chronic stress and those with major depressive disorder have been observed to be elevated [23].

This study aimed to investigate whether a relationship exists between anxiety levels and atopy, and dermographism and whether any neuroimmunological changes that occur in individuals with anxiety can affect skin prick test results and specific IgE measurements in patients.

## Materials and methods

Of the 202 subjects (112 women, 90 men) who consented in writing and participated in the study, 101 subjects with a Beck anxiety score of ≤7 points, which is the accepted threshold value, were included in the control group, and 101 subjects with a Beck anxiety score >7 were included in the study group. The cases were formed from hospital staff and patient relatives who gave written consent and agreed to participate in the study at the Mersin University Medical Faculty Hospital between November 2013 and March 2014. Skin prick test response, presence of phadiatop, and dermographism were evaluated in all participants.

To evaluate the presence of dermographism, pressures of 20 g/mm², 36 g/mm², and 60 g/mm² were applied to the paravertebral area of the upper back with a dermographometer (HTZ COMPANY HTZ Limited, Vulcan Way, New Addington, UK) within a 10-cm linear area. Thereafter, erythema and wheal reactions were evaluated. The mean of the widest and narrowest diameters of the wheal reaction was recorded with a 0.5-mm scale ruler. A wheal response greater than 2 mm was considered positive.

Blood samples taken for Phadiatop determinations were centrifuged at 4,500 rpm for 5 min, and the respective serums were then separated and stored in a freezer at −80°C. They were then collectively studied using the fluoro-allergo sorbent assay method (ImmunoCAP 100, Pharmacia, Stockholm, Sweden).

Between 09:00 and 12:00, skin prick tests were applied on the upper back region in 3-cm wide intervals. Before receiving the test, individuals were asked if they took calcium channel blockers, antidepressant drugs, antihistamines, corticosteroids, angiotensin-converting enzyme (ACE) inhibitors, H2 receptor blockers, and/or leukotriene receptor antagonists. Considering the half-life of these drugs in the patients who were scheduled for the skin prick test, they were first discontinued, and skin prick tests were performed in a week. The test areas were sanitized with ethyl alcohol and cotton and allowed to dry. Skin prick tests using 16 antigenic extracts were applied to the paravertebral area of the upper back (Table 1) using stallerpoint lancets (Stallergenes, Antony, France). After 15 and 20 min, responses for histamine + control and allergen extracts, respectively, were evaluated. A positive result was defined as a wheal reaction that was 2 mm more than the mean of the diagonal diameters of that formed by the negative control.

**Table 1:** Antigenic extracts used in skin prick test.

Skin prick test antigens
Positive control (histamine hydrochloride 10 mg/mL)
Negative control (temon)
Mites
Dermatophagoides farinea
Dermatophagoides pteronyssinus
Grain mix
Four-grain mix (barley, corn, oat, wheat)
Grass mix
Mixture of five grasses (fingergrass, weedgrass, meadow kelptail, meadow bluegrass, sweet spring grass)
Weed mix
Compositae (goldenhead, dandelion, daisy, pine tree)
Chenopodiaceae (white goosefoot, rough pigweed)
Tree pollen mix
Betulacea (alder, birch, hazelnut, hornbeam)
Fagaceae (beech, red oak, horse chestnut)
Oleaceae (ash, olive, privet)
Salicaceae (poplar, willow)
Fungal mixes
Aspergillus (fumigatus, niger, nidulans)
Cladosporium (clados poroides, herbarum)
Penicillium (digitatum, exponsum, notatum)
Animal product mix
Feather mix (duck, goose, chicken)

Beck anxiety scale

Beck anxiety scale evaluates the frequency of anxiety symptoms experienced by individuals. It is a self-assessment scale consisting of 21 items and scored between 0 and 3. The questions ask how much the feeling of distress had disturbed the individual during the past week. According to the test scores, the classifications are mild, moderate, and severe anxiety. The Turkish validity and reliability of the form developed by Beck et al. were performed by Ulusoy et al. (1998), and the Cronbach alpha coefficient of the scale was 0.93, which showed that the scale had sufficient reliability and validity [24-25].

For statistical analysis, the suitability of continuous variables for normal distribution was examined with the Shapiro-Wilk test. Continuous variables were summarized as median (minimum-maximum) since the distribution assumption was not provided. Categorical variables were summarized as numbers and percentages. The Chi-square test was used in the analysis of the crosstables, and binary ratio comparisons were made for meaningful results. The Mann-Whitney U test was used since the assumption of normal distribution was not provided in the comparison of two groups, and the Kruskal-Wallis test was used since the assumption of distribution was not provided in the comparison of more than two groups. Dunn's test, one of the post hoc tests, was used to find the source of the difference between the groups. p < 0.05 was accepted as the statistical significance level.

The ethics committee of Mersin University Faculty of Medicine approved the study (Approval 24/05/2013 and 2013/152).

As BAP TF DTB (NYA) 2013-3 TU coded project

It was supported by the Mersin University Scientific Research Projects Unit.

## Results

The demographic characteristics of the 202 patients aged 17-65 years are included in the study and are summarized in Table 2. The gender distribution among the groups was homogeneous (p = 0.078), and there was no statistically significant difference between the groups in terms of age (p = 0.900).

**Table 2:** Demographic characteristics of individuals in the study and control groups. SD, standard deviation

	Anxiety group Beck score: 8–15 (mild)	Anxiety group Beck score: 16–25 (moderate)	Anxiety group Beck score: 26–63 (severe)	Control group Beck score: 0–7 (no anxiety)	p
Age (mean year ± SD)	34.29 ± 13.456	37.28 ± 13.365	32.40 ± 13.644	34.54+12.190	0.900
Male/Female (n)	29 / 23	8 / 21	7 / 13	46 / 55	0.078

There were 41 (40.6%) and 47 patients (46.5%) that had positive skin test results in the study group with high anxiety levels and control group, respectively, which was not significantly different (p > 0.05). Phadiatop was positive in 34 (33.7%) and 42 patients (41.6%) in the study and control groups, respectively, which was also not statistically significant.

Dermographism was detected in 37 (20.8%) and 30 patients (15.8%) in the study and control groups, respectively. There was no statistically significant relationship between the groups in terms of dermographism, skin prick test, and Phadiatop results (p = 0.078, p = 0.395, and p = 0.245, respectively).

Dermographism subgroups

In the study and control groups, simple and symptomatic dermographism was found in 21 (20.8%) and 16 patients (15.8%), and 24 (23.8%) and 6 patients (5.9%), respectively. In terms of Beck scores, there was a statistically significant difference between the dermographism groups (p = 0.027). When the post hoc test was performed to determine the source of the difference, the results showed that the symptomatic group was statistically different from the other groups (p < 0.05). In patients with anxiety, symptomatic dermographism was found to be significantly higher (Table 3).

**Table 3:** Comparison of phadiatop, dermographism, and skin test results between the study group with anxiety and the control group.

		Anxiety group n (%)	Control group n (%)	Total	p
Dermographism	No	64 (63.4%)	71 (70.3%)	135 (66.8%)	0.078
	Simple	21 (20.8%)	24 (23.8%)	45 (22.3%)	
	Symptomatic	16 (15.8%)	6 (5.9%)	22 (10.9%)	
Skin prick test	Positive	41 (40.6%)	47 (46.5%)	88 (43.6%)	0.395
	Negative	60 (59.4%)	54 (53.5%)	114 (56.4%)	
Phadiatop	Positive	34 (33.7%)	42 (41.6%)	76 (37.6%)	0.245
	Negative	67 (66.3%)	59 (58.4%)	126 (62.4%)	

There was no statistically significant relationship between the groups in terms of skin prick test and Beck anxiety scores (p = 0.865) and Phadiatop (p = 0.630). On the contrary, there is a statistically significant relationship between the groups in terms of dermographism and Beck scores (p = 0.017) (Table 4).

**Table 4:** Comparison of groups according to Beck anxiety score.

			Anxiety group Beck score: 8–15 (mild)	Anxiety group Beck score: 16–25 (moderate)	Anxiety group Beck score: 26–63 (severe)	Control group Beck score: 0–7 (no anxiety)	p
Dermographism		No	38 (73.1)	12 (41.4%)	14 (70%)	71 (70.3%)	0.017
		Simple	8 (15.4)	11 (37.9%)	2 (10%)	24 (23.8%)	
		Symptomatic	6 (11.5)	6 (20.7%)	4 (20%)	6 (5.9%)	
Skin prick test	Positive		21 (40.4)	12 (41.4%)	8 (40%)	47 (46.5%)	0.865
	Negative		31 (59.6)	17 (58.6%)	12 (60%)	54 (53.4%)	
Phadiatop	Positive		19 (36.5)	9 (31%)	6 (30%)	42 (41.6%)	0.630
	Negative		33 (63.5)	20 (69%)	14 (70%)	59 (58.4%)	

In terms of histamine responses, when individuals with dermographism were compared with other groups, no statistical difference was found.

There was no statistically significant relationship between the groups in terms of their response to histamine applied as a positive control (Table 5).

**Table 5:** Comparison of wheal diameters as histamine 10 mg/mL response applied as positive control in skin prick test.

		Histamine responses median (min.-max.)	p
Groups	Anxiety group	7.5 (5–14.5)	0.720
	Control group	7.5 (4.5–12.5)	
Dermographism	No	7.5 (4.5–12.5)	0.578
	Simple	7.5 (5.25–14.5)	
	Symptomatic	7.5 (5.5–12)	
Skin prick test	Positive	7.5 (5–14.5)	0.095
	Negative	7.5 (4.5–12)	
Phadiatop	Positive	7.5 (4.5–14.5)	0.118
	Negative	7.5 (5–12)	

## Discussion

In our study, the effect of anxiety on dermographism and atopy tests was investigated. The results found no relationship between anxiety levels and atopy test responses; however, there was a statistically significant relationship between anxiety and symptomatic dermographism. Although a limitation of our study was that the skin tests could not be repeated in those with different anxiety levels, this situation provided an opportunity for the quantitative comparison between the groups by measuring the serologically specific IgE level in individuals.

While the incidence of atopic diseases has been increasing worldwide in recent years, few studies have been published that show the relationship between the nervous, endocrine, and immune systems in the same process.

Allergic diseases have both genetic and environmental components, and psychological comorbidity is an important aspect of these diseases; higher rates of anxiety have been reported in individuals with allergies than in non-allergic controls [26].

In 2007, Klokk et al. investigated the relationship between IgE levels and symptoms of anxiety and depression in an adult female population [27]. In the study, the role of IgE as a possible biological mechanism underlying the increase in the prevalence of anxiety and depression in atopic patients with asthma, rhinitis, and eczema was questioned, but no relationship between them was found.

Kiecolt-Glaser et al. investigated the effects of stress and anxiety on positive skin test responses in 28 young adults with allergic rhinitis. The results showed that laboratory stress increased allergen skin test responses (wheal sizes) in more anxious patients. Data from this study demonstrated that stress and anxiety can increase as well as prolong the symptoms of allergic rhinitis [28]; however, histamine (positive control) and negative control skin prick test responses were not affected by stress in the study. Unlike our study, the research investigated the effect of the acute stress response as a psychological factor on skin testing. In acute stressful situations, hormonal effects are more prominent and dominant. Stress-related immunological changes, particularly triggering the T helper 1 (TH1) to T helper 2 (TH2) switch, may increase allergic responses in susceptible individuals, and cortisol and norepinephrine can promote TH1 to TH2 transition [29]. The data on histamine responses in this study are consistent with the data in our study [28].

In Japan, a study conducted by Kimata determined that a negative mood caused by the sadness in cases where a drama video was shown decreased allergen wheal responses, whereas, in another study, it was determined that stress caused by insomnia (sleep deprivation) increased the skin’s wheal responses to the allergen [30-31]. Another study in Japan, where psychological factors such as positive affect were investigated, reported that laughing reduced allergic skin wheal responses in patients with atopic dermatitis, especially when the comedy video “Charlie Chaplin's Modern Times” or “Rowan Atkinson's The Best Bits of Mr. Bean” was shown [32]. In another Japanese study, the effects of two different composers’ works and music on atopy tests in atopic dermatitis patients who are allergic to latex were examined, and it was found that the reactions to allergic skin tests and levels of IgE antibodies in the blood decreased after they listened to a Mozart concert. No change was detected in the patient group who listened to Beethoven [33].

In a study investigating the relationship between depression as a psychological disorder and the skin prick test, Timonen et al. found that the number of patients with depression increased regardless if the skin prick result showed atopy in individuals with allergic symptoms, whereas a positive skin prick test result was not associated with depression in the absence of allergic symptoms [34]. On the other hand, Radosević-Vidacek et al. investigated the effects of exposure to stressful events, quality of life, and neuroticism on atopy. Their study included 145 patients; however, no relationship was found between psychosocial factors and atopy [35].

As a serological test, Phadiatop is a multi-specific IgE developed against common inhaled environmental allergens, and it is a valuable screening method for the diagnosis of allergic sensitivity. When the skin prick test is used as a reference in the general adult population, Phadiatop gives sufficiently accurate results when diagnosing allergic sensitization. Vidal et al. used the skin prick test results as a reference for the diagnosis of allergy sensitivity in 465 people and determined that the sensitivity, specificity, and positive predictive value of Phadiatop were 70.8%, 90.7%, and 72.6%, respectively [15]. In 100 patients clinically diagnosed with asthma and rhinitis, Eriksson found that the sensitivity, specificity, and positive predictive value of Phadiatop were 92%, 98%, 98%, respectively [36]. Traiyan et al. reported an excellent (94.2%) correlation between the skin prick test and Phadiatop in detecting aeroallergen sensitivity [37]. Based on previous reports showing its high sensitivity and high positive predictive value, the diagnostic value of Phadiatop has been confirmed. In various studies conducted in our country investigating the frequency of atopy, rates ranging from 3.5% and 48.6% have been reported [38-39]. In our study, the frequency of atopy was consistent with that of the general population, and no statistically significant difference was found between the results of the skin prick test and Phadiatop to diagnose atopy (p > 0.05). In addition, the sensitivity and specificity of Phadiatop were 70.1% and 93.81%, respectively, similar to those in the literature.

While the frequency of dermographism in our study was 33.2%, symptomatic dermographism was detected at a higher frequency in the anxiety group. This is consistent with data found in the literature, indicating that anxiety is one of the etiological factors in dermographism [13]. In terms of histamine responses, when individuals with dermographism were compared with other groups, no statistical difference was found. This result supports the role of neuromediators other than histamine in the dermographic response. When the groups were evaluated for polysensitization, sensitivity to more than one allergen was found in 19 cases in the anxiety group and in 21 cases in the control group, and there was no statistically significant difference between them.

These studies show that the relationship between psychosocial factors and atopy is complex, and many factors play a role in this interaction. In the case of prolonged and excessive stress, excessive stimulation of the hypothalamo-pituitary-adrenal axis leads to an increase in cell (Th1)-mediated/proinflammatory cytokines due to deficiencies in regulatory mechanisms and glucocorticoid signaling, and this leads to suppression of atopy due to Th2 responses. The increase in cortisol and epinephrine levels in the acute phase of the stress response in anxiety and inhibition of nuclear factor kappa B (and other intracellular proinflammatory signaling pathways) results in a decrease in cellular Th1 immune response and dominance of humoral (Th2) immune activation [21-22].

Many neurohormonal changes develop depending on the duration and severity of anxiety, which subsequently affects the immune system at different levels.

## Conclusions

Allergic diseases have both genetic and environmental components. Psychological comorbidity is an important aspect of these diseases; higher rates of anxiety have been reported in individuals with allergies than non-allergic control groups. Although the effect of anxiety on atopy test results is controversial, there was a statistically significant relationship between anxiety levels and dermographism in our study. Larger controlled-randomized studies with repeated skin prick tests at different anxiety levels are needed.
